# Efficacy and safety of bloodletting therapy for acute herpes zoster: a systematic review and meta-analysis

**DOI:** 10.3389/fneur.2025.1674245

**Published:** 2025-10-13

**Authors:** Danhui Wu, Yanbin Jiang, Qiqi Wu, Yibo Jin, Chenda Wang, Lihua Guan, Wei Shao

**Affiliations:** ^1^Department of Massage, Quzhou Hospital of Traditional Chinese Medicine, Quzhou, China; ^2^Department of Acupuncture and Moxibustion, Wenzhou Central Hospital, Wenzhou, China; ^3^School of Basic Medical Sciences, Zhejiang Chinese Medical University, Hangzhou, China; ^4^Department of Acupuncture and Moxibustion, Quzhou Hospital of Traditional Chinese Medicine, Quzhou, China

**Keywords:** herpes zoster, bloodletting therapy, systematic review, meta-analysis, postherpetic neuralgia, fire needle, cupping

## Abstract

**Background:**

The high incidence of acute herpes zoster (HZ) and limitations of conventional therapies in pain control necessitate exploration of complementary approaches. Bloodletting therapy (BLT), a traditional intervention with potential analgesic and immunomodulatory effects, has been increasingly applied in China, yet its efficacy remains controversial due to inconsistent clinical evidence.

**Methods:**

A systematic search was conducted in databases including PubMed, Embase, the Cochrane Library, MEDLINE, CNKI, the Wan Fang database, the Chinese clinical trial registry, and VIP Chinese Science from inception to June 2025 to identify randomized controlled trials (RCTs) comparing bloodletting therapy vs. pharmacotherapy in acute HZ (≤10 days post-eruption). Primary outcomes included Clinical efficacy rate, Visual Analog Scale (VAS), Skin lesion presentation, Incidence of PHN. Risk of bias was assessed using Cochrane RoB 2.0. Data were pooled via appropriate statistical methods (RevMan 5.4). PROSPERO registration: CRD420251110060.

**Results:**

A total of 16 RCTs involving 1,283 patients were included. Meta-analysis demonstrated that bloodletting therapy significantly improved clinical efficacy versus pharmacotherapy (OR = 4.51, 95%CI [2.89, 7.04], *p* < 0.00001). Furthermore, BLT achieved superior pain reduction, evidenced by lower VAS scores (MD = -1.57, 95%CI [−1.93, −1.21], *p* < 0.00001), and accelerated skin healing through shorter crust formation (MD = -1.58 days, 95%CI [−1.79, −1.38], *p* < 0.00001) and scab detachment times (MD = -2.53 days, 95%CI [−3.82, −1.23], *p* = 0.0001). Importantly, BLT reduced PHN incidence (OR = 0.23, 95%CI [0.15, 0.35], *p* < 0.00001). Regarding safety, adverse events were comparable between groups (OR = 0.66, 95%CI [0.31, 1.38], *p* = 0.26). Subgroup analyses suggested the superiority of fire needle techniques. Sensitivity analyses resolved high heterogeneity in some outcomes.

**Conclusion:**

Based on existing evidence predominantly, BLT may be associated with superior short-term outcomes in pain relief, skin lesion healing, and PHN prevention for acute herpes zoster compared to pharmacotherapy, and its safety profile appears acceptable. However, these findings must be interpreted with considerable caution due to the high risk of bias in the included studies and significant clinical heterogeneity. Moreover, the generalizability of the results is limited as all trials were conducted within a single region (China). Therefore, international, high-quality RCTs with standardized protocols and longer follow-ups are urgently needed to confirm these potential benefits and establish the long-term efficacy and safety of BLT.

## 1 Introduction

Herpes zoster (HZ) is an acute infectious disease caused by reactivation of the varicella-zoster virus (VZV) latent in sensory ganglia, characterized by unilateral dermatomal vesicular eruptions and severe neuralgia (1). Primary VZV infection manifests as chickenpox, after which the virus remains dormant in dorsal root ganglia. When cellular immunity declines, the virus reactivates and spreads along nerve pathways, causing neuritis and cutaneous lesions.

The global annual incidence of HZ is approximately 3–5‰ (2). As VZV-specific T-cell immunity wanes with age, individuals ≥50 years show significantly higher HZ incidence, exceeding 10‰ in those over 65 (3). A systematic review estimated the cumulative incidence between 2.9–19.5‰ (4), with advanced age, female sex, immunosuppression, and comorbidities being major risk factors (5, 6).

It is important to note that the introduction of recombinant zoster vaccine (RZV) has significantly reduced HZ incidence in vaccinated elderly cohorts in countries with high coverage. A systematic review indicates that the RZV (Shingrix) provides high effectiveness in real-world populations, with an approximate vaccine efficacy of 81%, and sustained protection throughout the first 2 years post-vaccination (7). However, epidemiological heterogeneity persists, and actual rates in regions without efficient reporting systems may be underestimated (8).

Postherpetic neuralgia (PHN), defined as persistent pain ≥90 days after rash healing (9), affects 5–30% of HZ patients (2). Reportedly, approximately 15.84% of HZ patients in the People’s Republic of China develop PHN (10). In contrast, the prevalence of PHN among HZ patients in Nepal is as high as 18.2% (11). Meanwhile, a study conducted in the United Kingdom found that about 9.55% of HZ patients progressed to PHN (12). It is noteworthy that developed countries exhibit lower rates of PHN compared to less developed regions, which is likely associated with higher vaccine coverage. The high cost of the vaccine has limited its widespread adoption across many regions.

This debilitating condition, often presenting with burning or electric-shock-like pain, may persist for months or years, severely compromising quality of life and mental health (13, 14). The global healthcare expenditure for HZ patients amounts to hundreds of millions of euros annually. In the United States, for instance, the average medical cost per hospitalized HZ case ranges from €9,041 to €23,220, with significantly higher expenses for elderly patients, immunosuppressed individuals, and those with complications (15, 16).

Current treatment focuses on antiviral therapy, pain control, and the PHN prevention (17), primarily using antivirals, analgesics, and neurotrophic agents (18). Antiviral medications such as acyclovir and valacyclovir are known to accelerate lesion healing, yet their analgesic effects are limited. Systematic reviews suggest that these agents contribute to only approximately 15% pain reduction, offering minimal benefit in cases of moderate-to-severe pain (19, 20). Despite timely antiviral treatment, PHN still develops in approximately 18.5% of patients aged 50 years and older (21). Analgesics including gabapentin and pregabalin provide at least 50% pain relief in only 30–50% of patients and are associated with frequent adverse effects such as dizziness, somnolence, gastrointestinal disturbances, as well as potential risks of addiction (22, 23). Although the recombinant zoster vaccine (RZV) reduces the risk of herpes zoster, its long-term efficacy beyond 10 years remains uncertain (24, 25). Furthermore, in resource-limited regions, its high cost significantly restricts accessibility.

Given HZ’s high incidence, PHN’s disabling nature, and suboptimal pain control with current therapies, exploring safe and effective complementary approaches is imperative. Bloodletting therapy (BLT), a traditional intervention with potential analgesic and immunomodulatory effects, warrants rigorous investigation.

With historical roots in China, India, and Mongolia (26), bloodletting employs specialized tools (three-edged needles, plum-blossom needles, fire needles) or cupping to release blood (27). The *Huangdi Neijing* (“Yellow Emperor’s Inner Canon”) established the principle of “removing stasis by pricking collateral vessels” to unblock meridian stagnation. Traditional Chinese Medicine (TCM) interprets acute HZ as “liver channel fire stagnation” and “spleen channel dampness-heat” congealing in skin and collaterals, causing erythema, vesicles, and pain. Following the TCM tenet of “venting fire through drainage,” bloodletting purportedly discharges pathogenic fire-toxins through blood release, potentially alleviating acute pain and preventing PHN progression (28, 29). By China’s Ming-Qing dynasties, it became a cornerstone of HZ management (30).

Recent years have witnessed a growing number of clinical studies investigating BLT for HZ, particularly RCTs conducted in China, which provide a foundation for systematic reviews. However, the methodological quality of many studies remains suboptimal, with limitations including small sample sizes, inadequate randomization and allocation concealment, challenges in blinding implementation, as well as inconsistent or non-standardized outcome measures. Furthermore, study findings demonstrate discrepancies—some report significant therapeutic benefits, particularly in alleviating acute-phase pain and cutaneous lesions (31), while others show no statistically significant difference compared to conventional pharmacotherapy (32). These inconsistent outcomes have engendered clinical controversies regarding the efficacy and safety of BLT for HZ among both practitioners and patients. Notably, there persists a lack of comprehensive and methodologically rigorous systematic reviews or meta-analyses specifically evaluating BLT for HZ, particularly those focusing on acute-phase pain management and PHN incidence.

To bridge this evidence gap, a systematic review and meta-analysis of RCTs assessing bloodletting therapy for acute HZ in both English and Chinese literature was performed. This study provides robust estimates of its effects on pain relief, lesion healing, PHN incidence, and safety through a quantitative synthesis of available data, thereby establishing an evidence-based foundation for clinical practice.

## 2 Methods

### 2.1 Search strategy

The following databases were searched to identify reports of relevant clinical trials from the inception to June 1, 2025. PubMed, Embase, Cochrane Library, MEDLINE, CNKI, Wan Fang database, Chinese clinical trial registry, and VIP Chinese Science. English or Chinese RCTs associated to HZ patients treating by BLT were included. The search strategy is shown in Appendix 1. In addition, ongoing or unpublished trials were also searched on clinical trial registry platforms, including the WHO International Clinical Trial Registry, and the Chinese Clinical Trial Registry. Inclusion and exclusion criteria were based on the PICO (Population, Intervention, Comparison, Outcome) method (33, 34).

### 2.2 Studies

RCTs regarding the effect of BLT for acute HZ patients were included in the review. The featured studies were written in either English or Chinese. Articles were eliminated if they were not RCTs or clinical trials. Researches were omitted if there was no matching complete paper published in a peer-reviewed journal or if no detailed data was supplied even after contacting the author.

### 2.3 Eligible criteria for study selection

#### 2.3.1 Types of studies

Published RCTs of the effects of BLT for acute HZ patients were included. Animal studies, case reports, and quasi-RCTs were exclude. Conference articles were excluded if the corresponding full text was not published or if specific data were not provided after contacting the authors. The included studies were published in English or Chinese.

#### 2.3.2 Types of participants

Eligible patients met the diagnostic criteria for herpes zoster, presented within 10 days of vesicle eruption, and had received no prior pharmacological or physical interventions.

#### 2.3.3 Types of interventions

The studies included in this review compared the effectiveness of BLT with pharmacotherapy. Techniques capable of directly achieving bloodletting, including three-edged needle, fire needle, and cutaneous acupuncture, were included. Interventions combined with other external treatment methods without directly inducing bleeding were excluded, such as regular acupuncture, electro acupuncture and phototherapy.

#### 2.3.4 Types of outcomes

The main outcomes could include one of these as follows, in terms of Clinical efficacy rate, Visual Analog Scale (VAS), Skin lesion presentation, Incidence of PHN.

### 2.4 Data collection and analysis

#### 2.4.1 Selection of studies

Two independent authors (DHW and WS) identified the articles by reading the title, abstract, and full text to including articles that meet the criteria. During the full-text selection process, divergences between the two review authors were resolved through discussion. When necessary, articles were arbitrated by a third author (QQW). These studies were imported into EndNote X9 (Clarivate Analytics, Philadelphia, PA, USA).

#### 2.4.2 Data extraction and management

The two review authors (DHW and WS) independently extracted data from all included studies. Differences were thrashed out with the help of a third author (QQW). The extracted data were recorded into an Excel spreadsheet designed to obtain the following detailed trial information: study author, publication year, sample size, characteristic of participants, interventions, course of treatment and outcome measure. Extract the mean differences (MDs) of the results reported for continuous variables. Appropriate conversions are applied when results are reported as median, quartile range or mean, and 95% CIs.

#### 2.4.3 Dealing with missing data

If the data is only provided in chart form instead of numerical form, we planned to estimate the MDs from the chart. The corresponding authors were contacted in cases of completely missing data. If no response was received or the missing data could not be provided, all available data were included in the analysis. If the available data is insufficient for analysis, we presented descriptive data in the article.

#### 2.4.4 Assessment of risk of bias

The risk of bias will be assessed by two independent reviewers (YBJ and CDW) using The Cochrane Handbook (35). Any discrepancies will be resolved through discussion with a third researcher (LHG). The Cochrane Handbook for Systematic Reviews of Interventions worked according to the following criteria: random sequence generation, allocation concealment, blinding of participants and personnel, blinding of outcome assessment, incomplete outcome data, selective reporting, and other forms of bias. These items are estimated with ‘Low’ or ‘High’ or ‘Unclear’.

#### 2.4.5 Measures of treatment effect

Continuous variable outcomes were analyzed using MD. When *p* < 0.05, it was considered statistically significant. For results using different units, we used MD with a 95% CI to represent the aggregate estimate. We intuitively assess heterogeneity based on *I* (2) value. When the heterogeneity is greater than 50%, the random effects model is adopted, otherwise the fixed effects model is adopted (36, 37). The DerSimonian–Laird estimator was used for the random-effects model to account for between-study variance in heterogeneity. Begg’s funnel plot and Egger’s linear regression test were applied to evaluate publication bias. Subgroup analyses were pre-specified to explore potential sources of heterogeneity including variations in bloodletting techniques. These were planned *a priori* in the study protocol (PROSPERO: CRD420251110060). Data compilation and statistical analysis were performed using Review Manager 5.4.

## 3 Result

### 3.1 Search and selection

**Figure 1:**
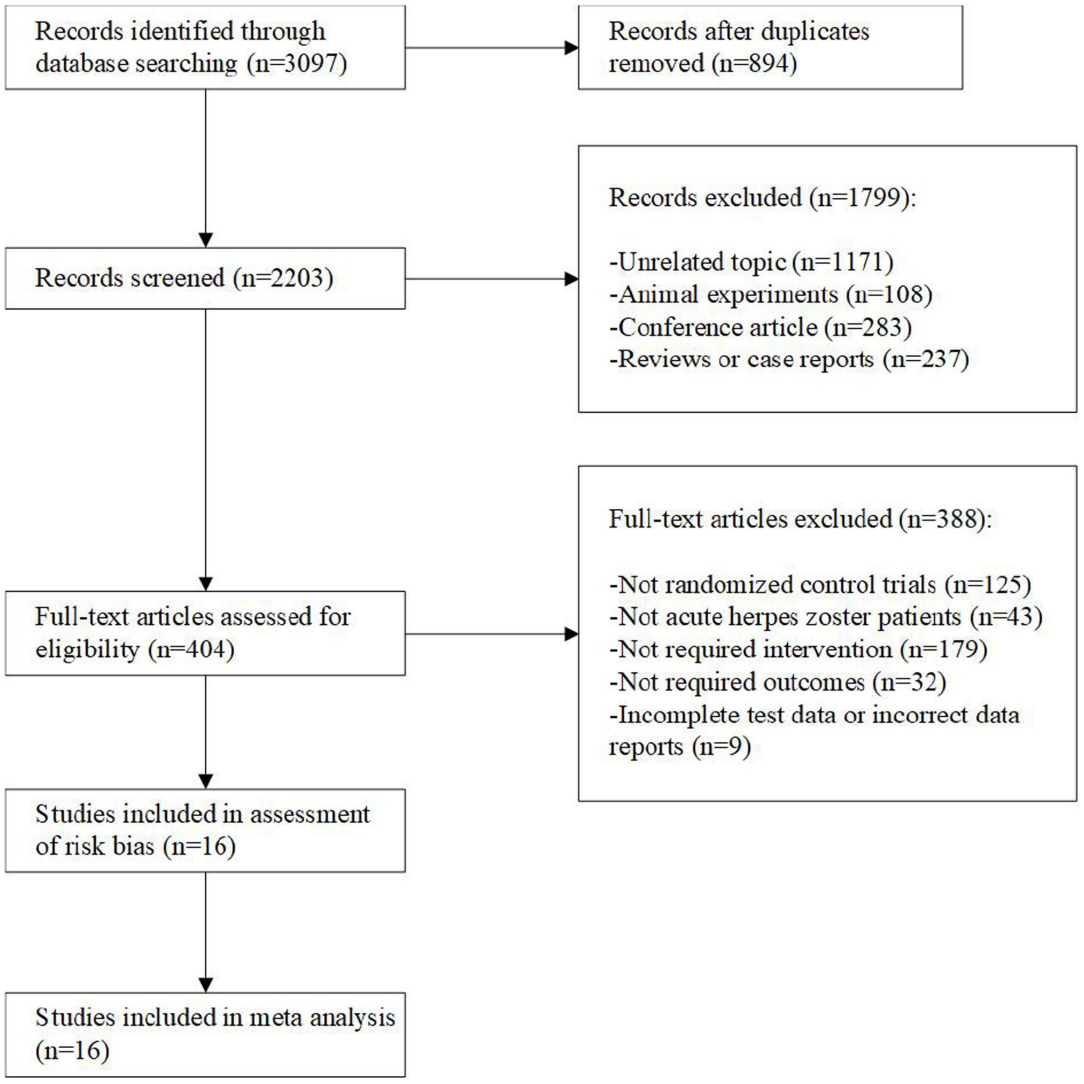
Flowchart of the process used to retrieve relevant articles.

The search strategy yielded 3,097 publications. 2,203 articles remained after eliminating duplicate studies with Endnote. 404 studies were retrieved for evaluation by screening titles and abstracts to eliminate irrelevant topics, animal experiments, conference papers, reviews. By further reading the full text, 16 studies were finally identified for systematic review, with a total of 1,283 HZ patients. The screening process is shown below (Figure 1).

### 3.2 Study characteristics

Basic characteristics of included studies in the systematic review are summarized in Table 1. All the included researches were from China. The mean age arranged from 40 years (SD9.6) to 65 years (SD11.0), and all studies included both male and female participants. In terms of the intervention measures in the experimental group, 10 studies (31, 32, 38–45) used fire needle to achieve bloodletting, five studies (46–50) chose three-edged needle for bloodletting, and one study (51) chose cutaneous needle (plum blossom needle) for percussion bloodletting. The minimum course of BLT intervention is 7 days and the maximum is 4 weeks.

**Table 1:** The characteristics of the randomized controlled trials (RCTs) included in this meta-analysis.

Included trials	Intervention and treatment		Sample and characteristics (male/female, age, disease duration)			Outcomes
	Trial	Control	Trial	Control		
Xu et al. (38)	Fire needling bloodletting and cupping Duration: 14 days	Pharmacological (valacyclovir, mecobalamin, vitamin B1) Duration: 14 days	30 (M:13, F17); AGE: (mean: 49.35 ± 12.32 years); Disease duration: (mean: 4.15 ± 1.15 days)	30(M:16, F:14); AGE: (mean: 50.31 ± 11.36 years); Disease duration: (mean: 3.92 ± 1.56 days)		Clinical efficacy rate, VAS, Skin lesions, SP, Adverse reaction
Cao et al. (46)	Three-edged needle bloodletting with cupping Duration: 14 days-28 days	Pharmacological (cobamamide, vitamin B1, vitamin B12) Duration: 12 days	33(M:14, F:19); AGE: (mean: 56.81 ± 11.76 years); Disease duration: (mean: 4.21 ± 1.41 days)	33 (M:13, F:20); AGE: (mean: 57.12 ± 12.46 years); Disease duration: (mean: 4.18 ± 1.65 days)		Clinical efficacy rate, VAS, Symptoms score, Adverse reaction
Zeng et al. (31)	Fire needling bloodletting and cupping Duration: 10 days	Pharmacological (valacyclovir, vitamin B1) Duration: 10 days	40(M:16, F:24); AGE: (mean: 50 ± 13 years); Disease duration: (mean: 3.8 ± 2.1 days)	40(M:21, F:19); AGE: (mean: 46 ± 17 years); Disease duration: (mean: 3.2 ± 1.6 days)		Clinical efficacy rate, VAS, Skin lesions, Symptoms score, Incidence of PHN, Adverse reaction, Th17, Treg, IL-10, IL-7
Fang et al. (39)	Fire needling bloodletting and cupping Duration: 7 days	Pharmacological (famciclovir) Duration: 7 days	39(M:19, F:20); AGE: (mean: 56.51 ± 17.36 years); Disease duration: 1–6 days	37(M:11, F:26); AGE: (mean: 58.76 ± 16.00 years); Disease duration: 1–6 days		Clinical efficacy rate, VAS, Skin lesions, Incidence of PHN, Adverse reaction, Brief McGill, SF-36
Gai et al. (47)	Three-edged needle bloodletting with cupping Duration: -d	Pharmacological (acyclovir, mecobalamin) Duration: 14 days	50(M:26, F:24); AGE: (mean: 58.67 ± 3.57 years); Disease duration: -	50(M:27, F:23); AGE: (mean: 59.01 ± 3.48 years); Disease duration: -		Crust Formation time, VAS, PSQI, DLQI, HAMA
He et al. (40)	Fire needling bloodletting and cupping Duration: 7 days	Pharmacological (famciclovir) Duration: 7 days	34(M:15, F:19); AGE: (mean: 58.26 ± 17.30 years); Disease duration: (mean: 4.08 ± 1.67 days)	33(M:9, F:24); AGE: (mean: 60.03 ± 15.24 years); Disease duration: (mean: 4.83 ± 1.11 days)		Clinical efficacy rate, VAS, Skin lesions, Incidence of PHN, Adverse reaction, Brief McGill, Pain relief time
Li et al. (41)	Fire needling bloodletting and cupping Duration: 10 days	Pharmacological (valacyclovir, vitamin B1) Duration: 10 days	30(M:9, F:21); AGE: (mean: 49.37 ± 10.07 years); Disease duration: (mean: 3.83 ± 2.07 days)	30(M:8, F:22); AGE: (mean: 46.70 ± 11.25 years); Disease duration: (mean: 3.23 ± 1.59 days)		Clinical efficacy rate, Skin lesions, Incidence of PHN, IL-10, IL-7
Xing et al. (51)	Cutaneous needle bloodletting combined with cupping Duration: 14 days	Pharmacological (valacyclovir, foscarnet sodium and sodium chloride) Duration: 14 days	28(M:14, F:14); AGE: (mean: 40.02 ± 19.62 years); Disease duration: (mean: 13.13 ± 8.45 days)	28(M:15, F:13); AGE: (mean: 42.50 ± 18.32 years); Disease duration: (mean: 10.51 ± 7.25 days)		Clinical efficacy rate, VAS, Incidence rate of PHN
Pan et al. (48)	Three-edged needle bloodletting with cupping Duration: 14 days	Pharmacological (famciclovir) Duration: 14 days	32(M:18, F:14); AGE: (mean: 48.62 ± 12.03 years); Disease duration: (mean: 3.62 ± 2.03 days)	32(M:19, F:13); AGE: (mean: 49.84 ± 11.65 years); Disease duration: (mean: 3.84 ± 1.65 days)		Clinical efficacy rate, VAS, Skin lesions, Symptoms score, Pain relief time
Song et al. (49)	Three-edged needle bloodletting with cupping Duration: 7 days	Pharmacological (acyclovir) Duration: 7 days	80(M:50, F:30); AGE: 30–70 years; Disease duration: 3–7 days	70(M:40, F:30); AGE: 27–69 years; Disease duration: 3–7 days		Clinical efficacy rate, Incidence rate of PHN
Tian et al. (42)	Fire needling bloodletting and cupping Duration: 10 days	Pharmacological (valacyclovir, vitamin B1) Duration: 10 days	51(M:16, F:35); AGE: (mean: 45.82 ± 12.23 years); Disease duration: (mean: 2.37 ± 1.12 days)	51(M:19, F:32); AGE: (mean: 45.36 ± 12.02 years); Disease duration: (mean: 2.32 ± 1.08 days)		Incidence rate of PHN, Th17, Treg, Th17/Treg
Zhang et al. (50)	Three-edged needle bloodletting with cupping Duration: 7 days	Pharmacological (acyclovir, mecobalamin) Duration: 14 days	32(M:11, F:21); AGE: (mean: 60.8 ± 10.3 years); Disease duration: (mean: 4.11 ± 1.09 days)	31(M:10, F:21); AGE: (mean: 65.5 ± 11.0 years); Disease duration:(mean: 4.27 ± 1.16 days)		VAS, Adverse reaction, Number of herpes, DLQI, SAS
Wang et al. (43)	Fire needling bloodletting and cupping Duration: 9 days	Pharmacological (famciclovir) Duration: 9 days	55(M:-, F:-); AGE:-; Disease duration:-d	54(M:-, F:-); AGE:-; Disease duration:-d		Clinical efficacy rate, Incidence rate of PHN, Adverse reaction, Symptoms score
Wen et al. (44)	Fire needling bloodletting and cupping Duration: 10 days	Pharmacological (acyclovir, prednisone, acyclovir cream) Duration: 14 days	50(M:33, F:17); AGE: (mean: 48.12 ± 11.23 years); Disease duration: (mean: 3.25 ± 1.45 days)	50(M:31, F:19); AGE: (mean: 47.58 ± 10.14 years); Disease duration: (mean: 3.27 ± 1.43 days)		Clinical efficacy rate, Skin lesions, Incidence rate of PHN, Pain relief time
Xu et al. (32)	Fire needling bloodletting and cupping Duration: 10 days	Pharmacological (valacyclovir, vitamin B1, gabapentin) Duration: 10 days	30(M:12, F:18); AGE: (mean: 57.83 ± 12.67 years); Disease duration: (mean: 3.33 ± 3.70 days)	30(M:7, F:23); AGE: (mean: 58.67 ± 12.59 years); Disease duration: (mean: 3.00 ± 1.48 days)		Clinical efficacy rate, VAS, Incidence rate of PHN, Adverse reaction, Th17, Treg, Th17/ Treg, Pain relief time
Zhang et al. (45)	Fire needling bloodletting and cupping Duration: 10 days	Pharmacological (valacyclovir, mecobalamin, gabapentin) Duration: 10 days	36(M:16, F:20); AGE: (mean: 51.19 ± 13.17 years); Disease duration: (mean: 4.11 ± 1.43 days)	34(M:14, F:20); AGE: (mean: 49.85 ± 13.42 years); Disease duration: (mean: 4.06 ± 1.23 days)		Clinical efficacy rate, VAS, Skin lesions, Incidence rate of PHN, Adverse reaction, Symptoms score, PSQI

VAS, Visual Analog Scale; Brief McGill, Short-form McGill Pain Questionnaire; SF-36, 36-Item Short Form Health Survey; PSQI, Pittsburgh Sleep Quality Index; DLQI, Dermatology Life Quality Index; HAMA, Hamilton Anxiety Rating Scale; SAS, Self-Rating Anxiety Scale.

In the control groups, pharmacological therapies were uniformly administered, primarily consisting of antiviral agents, combined with neurotrophic drugs, analgesics, or corticosteroids. All 15 studies (31, 32, 38–45, 47–51) utilized antiviral medications, nine studies (31, 32, 38, 41, 42, 45–47, 50) included neurotrophic drugs, two studies (32, 45) employed analgesic drugs, and one study (44) incorporated corticosteroids, with treatment courses ranging from seven to 14 days.

For outcome measures, 13 studies (31, 32, 38–41, 43–46, 48, 49, 51) reported effective rates, 10 studies (31, 32, 38–40, 43–48, 51) provided VAS scores, 10 studies (31, 32, 39–45, 49, 51) documented PHN incidence, six studies (31, 38, 39, 41, 44, 48) reported vesicle cessation time, eight studies (31, 38–41, 44, 47, 48) recorded crust formation time, and four studies (31, 38, 39, 41) reported scab detachment time. Laboratory-related indicators were provided in five studies (31, 32, 38, 41, 42). Adverse events were documented in seven studies (31, 32, 38, 40, 43, 45, 46). Quality of life related scores were reported in three studies (39, 47, 50), while PSQI scores (45, 47), Brief McGil scores (39, 40) and anxiety-related scores (47, 50) were each reported in two studies. The baseline characteristics of the included studies were comparable, as no significant differences (*p* > 0.05) were observed between intervention and control groups in terms of disease duration, age, or gender distribution.

### 3.3 Risk of bias in the included studies

**Figure 2:**
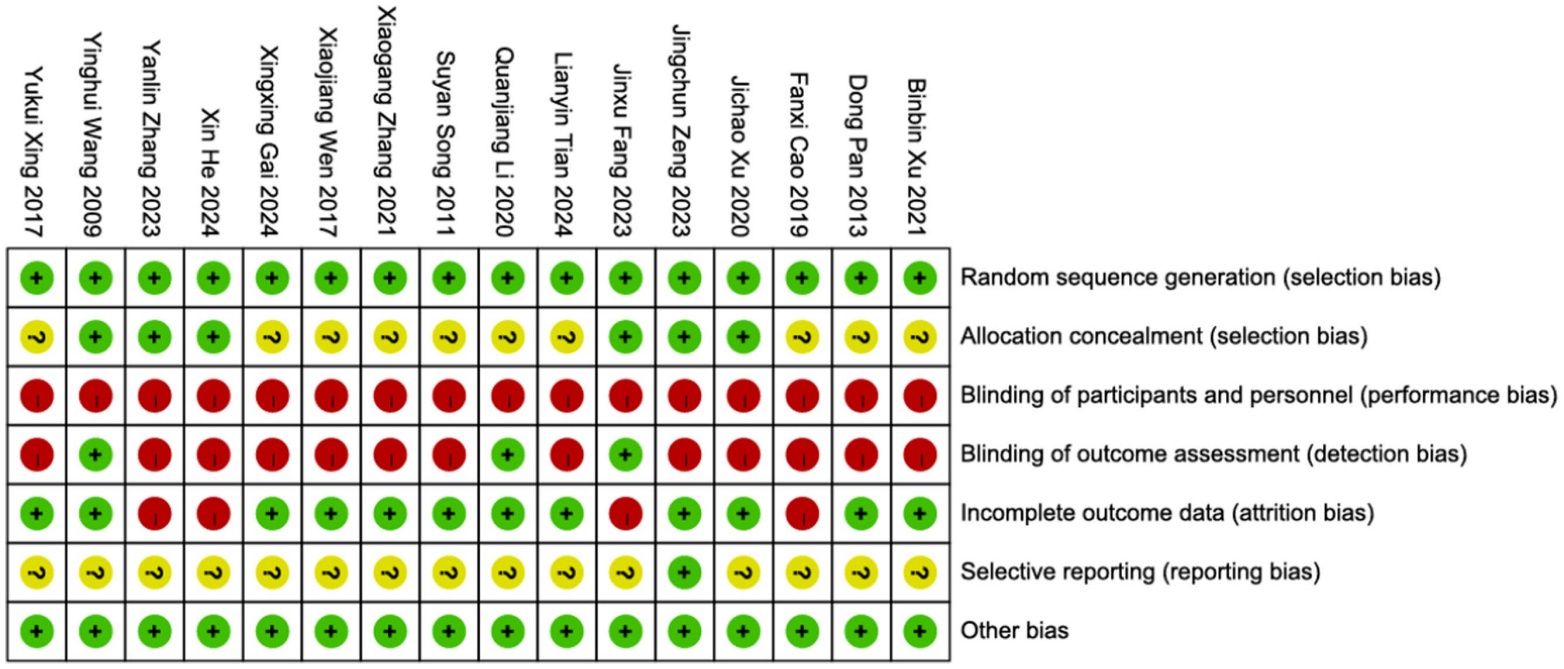
An evaluation of risk bias in the included RCTs.

The risk of bias was assessed using the Cochrane Risk of Bias Tool (Handbook 5.3.0), as detailed in Figure 2.

Randomization: Since we excluded quasi-randomized or semi-randomized studies, all included trials were rated as low risk in random sequence generation. Among them, 13 studies used a random number table, two studies employed central randomization, and one study utilized computer-generated randomization. Allocation concealment: Six studies described the allocation concealment process. Of these, two studies achieved concealment through central randomization, one study used the Lnkmed research platform, and three studies implemented opaque sealed envelopes. Blinding: Due to the nature of the interventions, none of the 16 included studies achieved double-blinding. However, three studies applied assessor blinding (outcome evaluators were masked). Attrition bias: Four studies were considered at risk due to incomplete outcome data, as they reported participant dropouts but did not perform intention-to-treat (ITT) analysis. The remaining studies had complete outcome data with a low risk of bias. Selective reporting: One study was confirmed to be free of selective reporting bias, while for the remaining 15 studies, it could not be determined whether all pre-specified outcome measures had been reported as planned. Additionally, no other sources of bias were identified.

### 3.4 Effective rate

**Figure 3:**
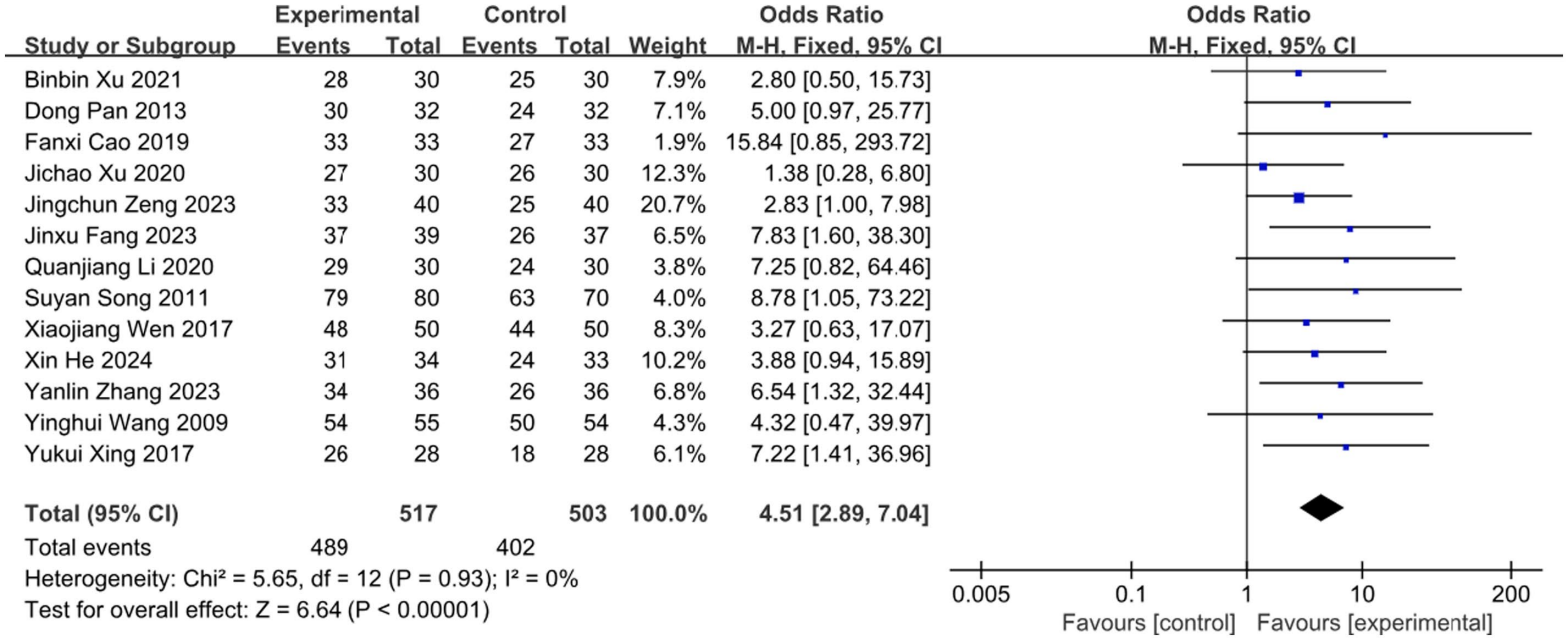
Forest plot and meta-analysis of the clinical efficacy rate of BLT in the treatment of HZ.

**Figure 4:**
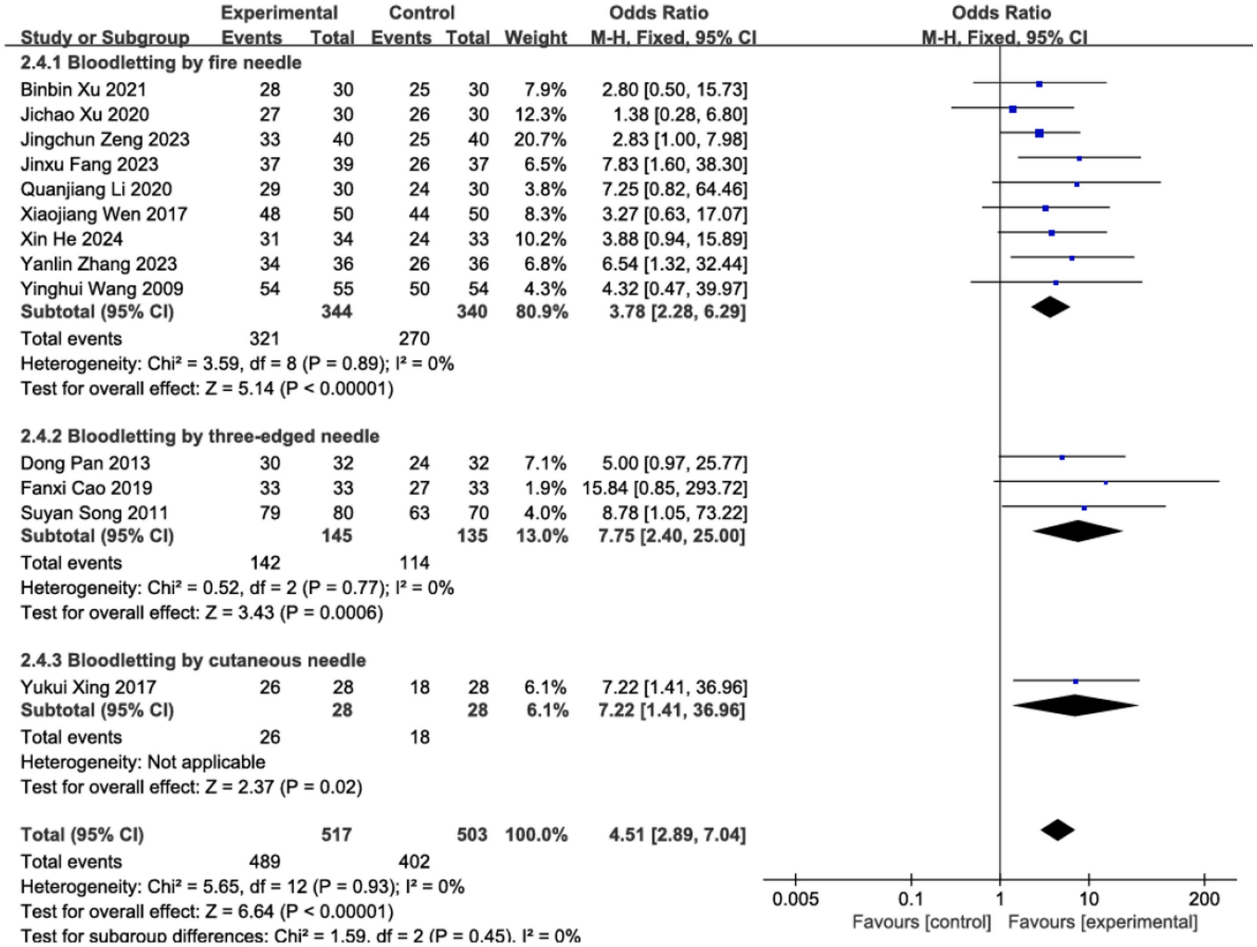
Subgroup analysis of different methods for bloodletting.

The clinical efficacy of BLT for acute HZ was examined across 13 RCTs, which included a total of 1,020 participants. The meta-analysis demonstrated that BLT was significantly superior to pharmacological treatment in terms of efficacy (OR = 4.51; 95% CI: [2.89, 7.04]; *p* < 0.00001; Figure 3). No significant heterogeneity was observed among the studies (*I*^2^ = 0%, *p* = 0.93). Given the variations in bloodletting methods across trials, subgroup analyses were conducted based on different intervention techniques, and the results remained consistent with the primary analysis. Notably, fire needle interventions emerged as the most promising approach, evidenced by significant pain reduction and lesion resolution. (Figure 4). Exploratory subgroup analyses suggested potential differences, but these results are hypothesis-generating and require validation.

### 3.5 VAS

**Figure 5:**
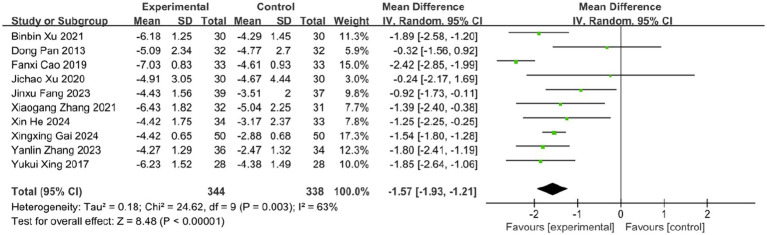
Meta-analysis of VAS data.

**Figure 6:**
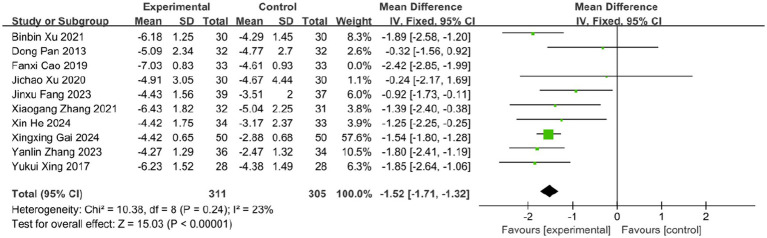
Meta-analysis of VAS data (after adjustment for heterogeneity).

Pain is one of the most prominent symptoms of acute HZ. In this review, 10 studies evaluated the effect of BLT on pain intensity using the VAS, involving 682 participants. Due to substantial heterogeneity (*I*^2^ = 63%, *p* = 0.003), a random-effects model was applied. The results indicated that BLT significantly reduced VAS scores compared to drug therapy (MD: -1.57; 95% CI: [−1.93, −1.21]; *p* < 0.00001; Figure 5). Importantly, this magnitude of reduction exceeds the established minimal clinically important difference (MCID) of 1.0–1.5 points for acute pain on the VAS scale (52, 53), suggesting that the improvement is not only statistically significant but also clinically meaningful for patients. After excluding the study by Fanxi Cao, heterogeneity decreased significantly (*I* (2) = 23%, *p* = 0.24), suggesting this study was the primary source of heterogeneity. Heterogeneity may stem from variability in study protocols, including differences in bleeding volume, treatment sessions, and frequency, as well as blinding and outcome assessment methods. The study by Fanxi Cao, which used a larger bleeding volume and had a higher risk of bias, may further contribute to heterogeneity due to limited robustness and generalizability. Future high-quality trials with standardized protocols and bloodletting volume comparisons are needed to clarify these factors. The meta-analysis excluding this study still supported the superiority of BLT (MD: −1.52; 95% CI: [−1.71, −1.32]; *p* < 0.00001; Figure 6).

### 3.6 Skin lesion outcomes

#### 3.6.1 Time to vesicle cessation

**Figure 7:**
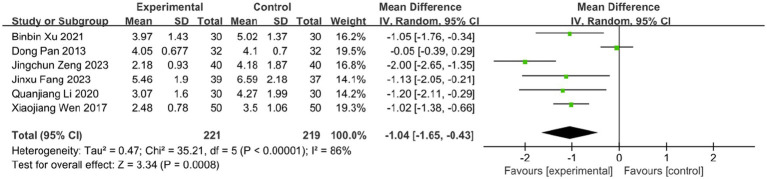
Meta-analysis of time to vesicle cessation data.

**Figure 8:**
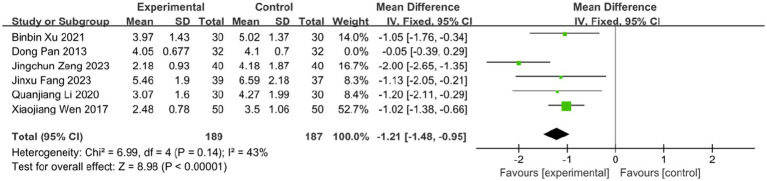
Meta-analysis of time to vesicle cessation data (after adjustment for heterogeneity).

The time to vesicle cessation is a critical clinical indicator for monitoring HZ progression. Six studies assessed the effect of BLT on this outcome, including 440 participants. Due to high heterogeneity (*I^2^* = 86%, *p* < 0.00001), a random-effects model was used. The results showed that BLT significantly shortened the time to vesicle cessation compared to drug therapy (MD: -1.04; 95% CI: [−1.65, −0.43]; *p* = 0.0008; Figure 7). After excluding the study by Dong Pan, heterogeneity decreased (*I^2^* = 43%, *p* = 0.14). Potential reasons for the outlier effect included the study’s small sample size, insufficient statistical power, and potential bias due to inadequate blinding. The meta-analysis excluding this study still confirmed the efficacy of BLT (MD: -1.21; 95% CI: [−1.48, −0.95]; *p* < 0.00001; Figure 8).

#### 3.6.2 Time to crust formation

**Figure 9:**
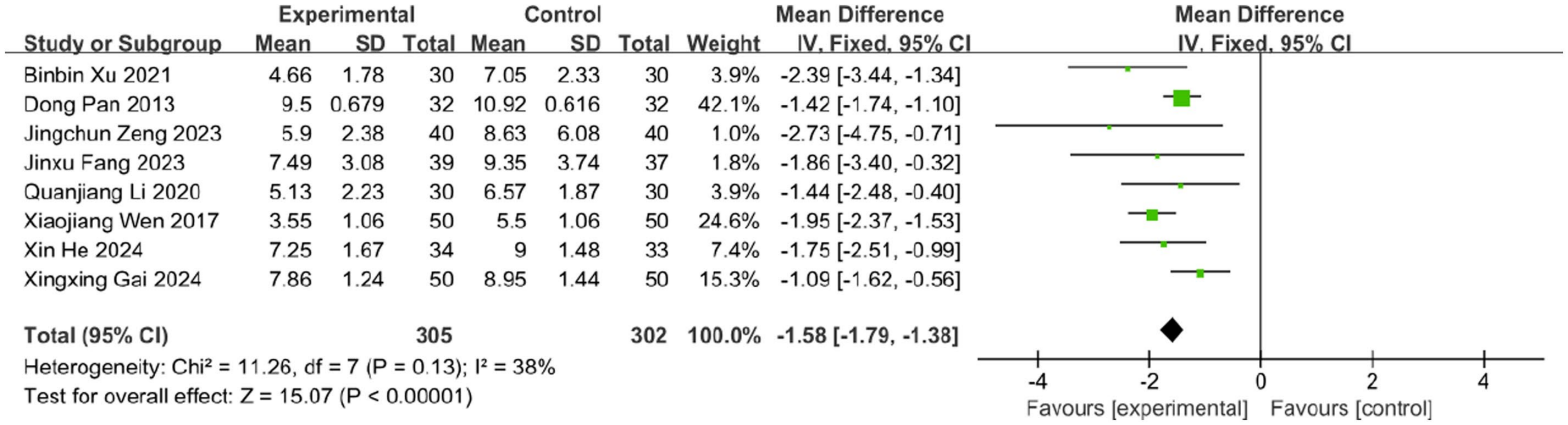
Meta-analysis of time to crust formation data.

Eight studies evaluated the effect of BLT on the time to crust formation, involving 607 participants. Given acceptable heterogeneity (*I^2^* = 38%, *p* = 0.13), a fixed-effects model was applied. The results demonstrated that BLT significantly reduced the time to crust formation compared to drug therapy (MD: -1.58; 95% CI: [−1.79, −1.38]; *p* < 0.00001; Figure 9).

#### 3.6.3 Time to scab detachment

**Figure 10:**
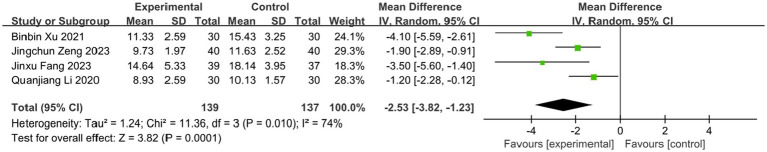
Meta-analysis of time scab detachment data.

**Figure 11:**
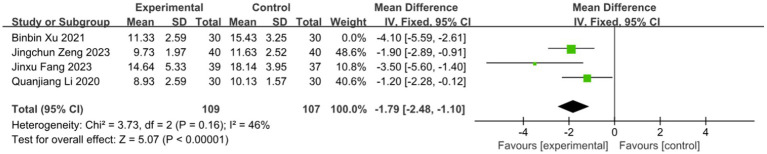
Meta-analysis of time scab detachment data (after adjustment for heterogeneity).

Four studies assessed the effect of BLT on the time to scab detachment, including 276 participants. Due to substantial heterogeneity (*I^2^* = 74%, *p* = 0.010), a random-effects model was used. The results indicated that BLT significantly shortened the time to scab detachment (MD: -2.53; 95% CI: [−3.82, −1.23]; *p* = 0.0001; Figure 10). The study by Binbin Xu exhibited a notably larger standard deviation (SD = −4.10) compared to others. After exclusion, heterogeneity decreased (*I^2^* = 46%, *p* = 0.16). Similar to previous outliers, this study had a small sample size and potential bias from inadequate blinding. The meta-analysis excluding this study still supported the significant reduction in scab detachment time with BLT (MD: -1.79; 95% CI: [−2.48, −1.10]; *p* < 0.00001; Figure 11).

### 3.7 Incidence of PHN

**Figure 12:**
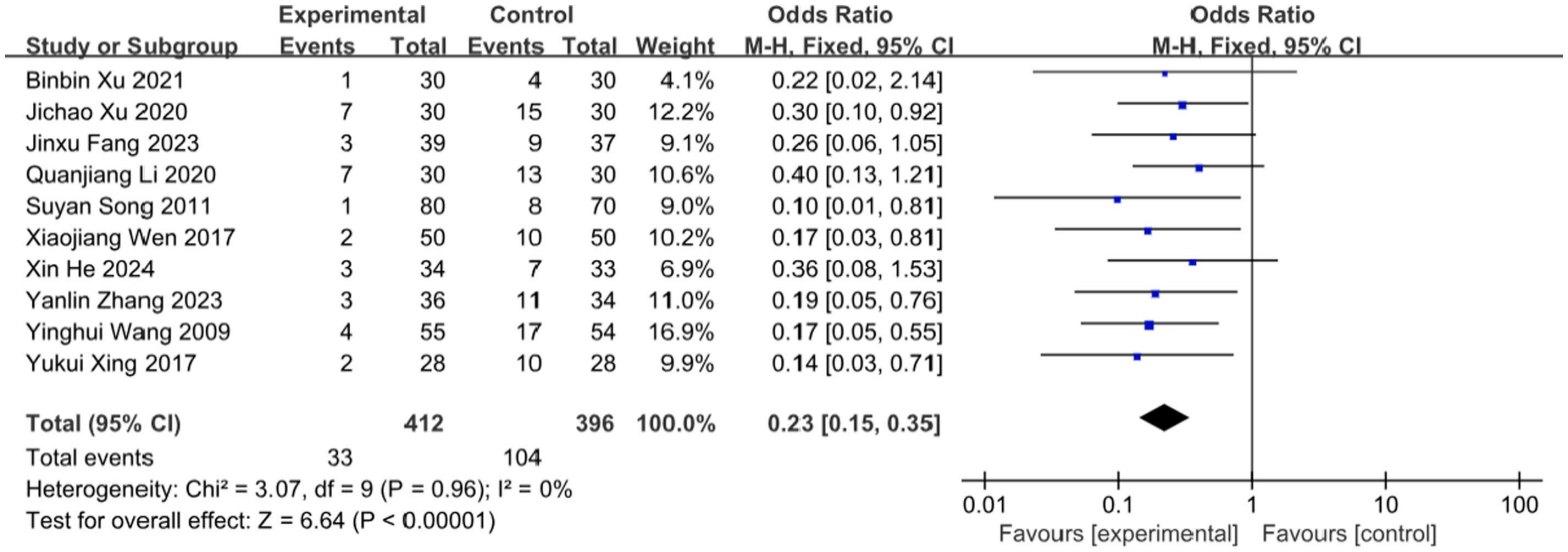
Meta-analysis of incidence of PHN.

Postherpetic neuralgia (PHN), the most severe complication of herpes zoster (HZ), significantly impairs patients’ quality of life and mental health. Ten studies in this review evaluated the effect of BLT on PHN incidence, involving 808 participants. The meta-analysis demonstrated that BLT significantly reduced PHN incidence compared to pharmacological treatment (OR = 0.23; 95% CI: [0.15, 0.35]; *p* < 0.00001; Figure 12). No significant heterogeneity was observed among studies (*I^2^* = 0%, *p* = 0.96). However, it is important to note that the assessment of PHN in the included studies was based on a relatively short follow-up period (typically ≤3 months). While this demonstrates a significant short-term preventive effect, longer-term follow-up beyond the 90-day diagnostic threshold is needed to confirm the sustained efficacy of BLT in preventing established PHN.

### 3.8 Adverse events

Among the 16 included RCTs, 7 studies reported adverse events (AEs), totaling 28 cases. In the bloodletting group (four studies), reported AEs included: Needling syncope (4 cases, 1.55%), Subcutaneous hemorrhage (4 cases, 1.55%), Excessive bleeding (1 case, 0.39%), Local skin damage (1 case), Minor burns (1 case).

In the control group (seven studies), AEs comprised: Gastrointestinal discomfort (9 cases, 3.52%), Palpitations (1 case, 0.39%), Headache (1 case, 0.39%), Dizziness (2 cases, 0.78%), Diarrhea (1 case, 0.39%), Unspecified events (3 cases, 1.17%).

**Figure 13:**
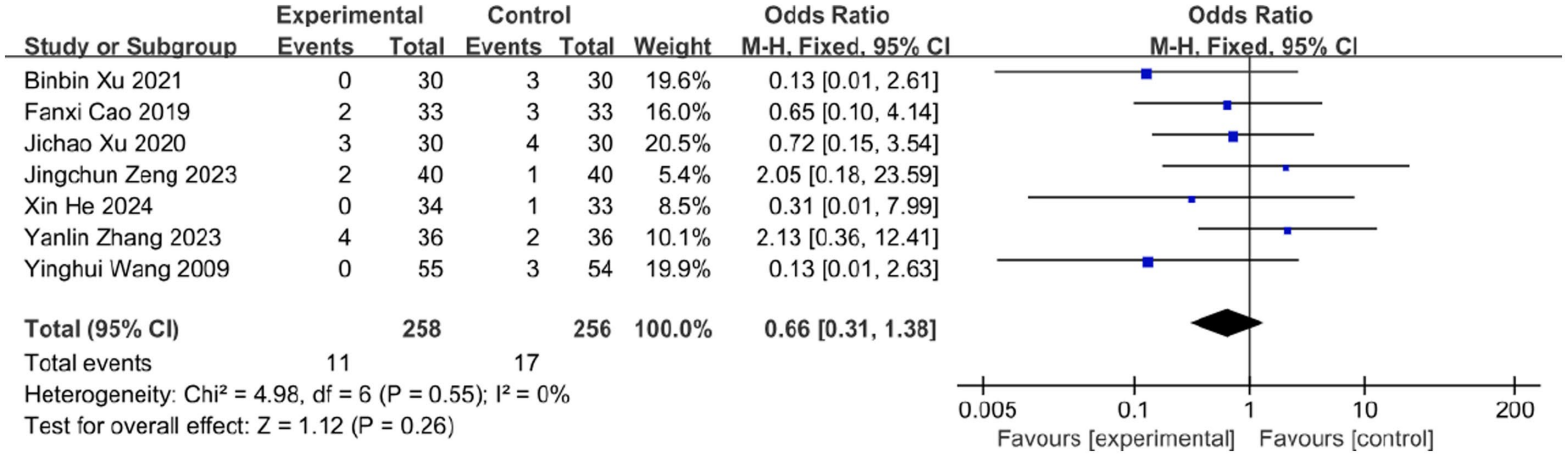
Meta-analysis of adverse events.

Meta-analysis showed a numerically higher AE rate in controls, but the difference was not statistically significant (OR = 0.66; 95% CI: [0.31, 1.38]; *p* = 0.26; Figure 13), with no heterogeneity (*I^2^* = 0%, *p* = 0.55). Both therapies have the potential to cause serious adverse effects, such as burns and severe allergic reactions, which are not mentioned by included studies. This absence of reports may be attributed to the limited number of studies available for inclusion, along with the possibility of underreported complications in clinical practice. Therefore, the safety profile of bloodletting therapy remains to be further validated.

### 3.9 TH17/Treg

**Figure 14:**
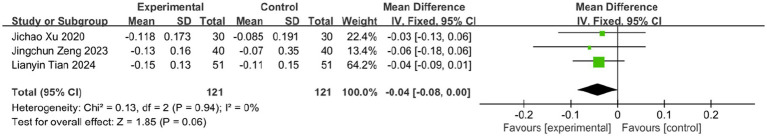
Meta-analysis of TH17/Treg.

The TH17/Treg balance critically influences HZ progression, neuroinflammation severity, and PHN risk. Clinical studies indicate elevated peripheral TH17/Treg ratios in PHN patients versus recovered individuals. Three studies (*n* = 242) assessed bloodletting’s effect on TH17/Treg levels. While bloodletting showed a trend toward greater improvement than drug therapy, the difference was nonsignificant (MD = −0.04; 95% CI: [−0.08, 0.00]; *p* = 0.06; Figure 14), with no heterogeneity (*I^2^* = 0%, *p* = 0.94).

### 3.10 Publication bias

**Figure 15:**
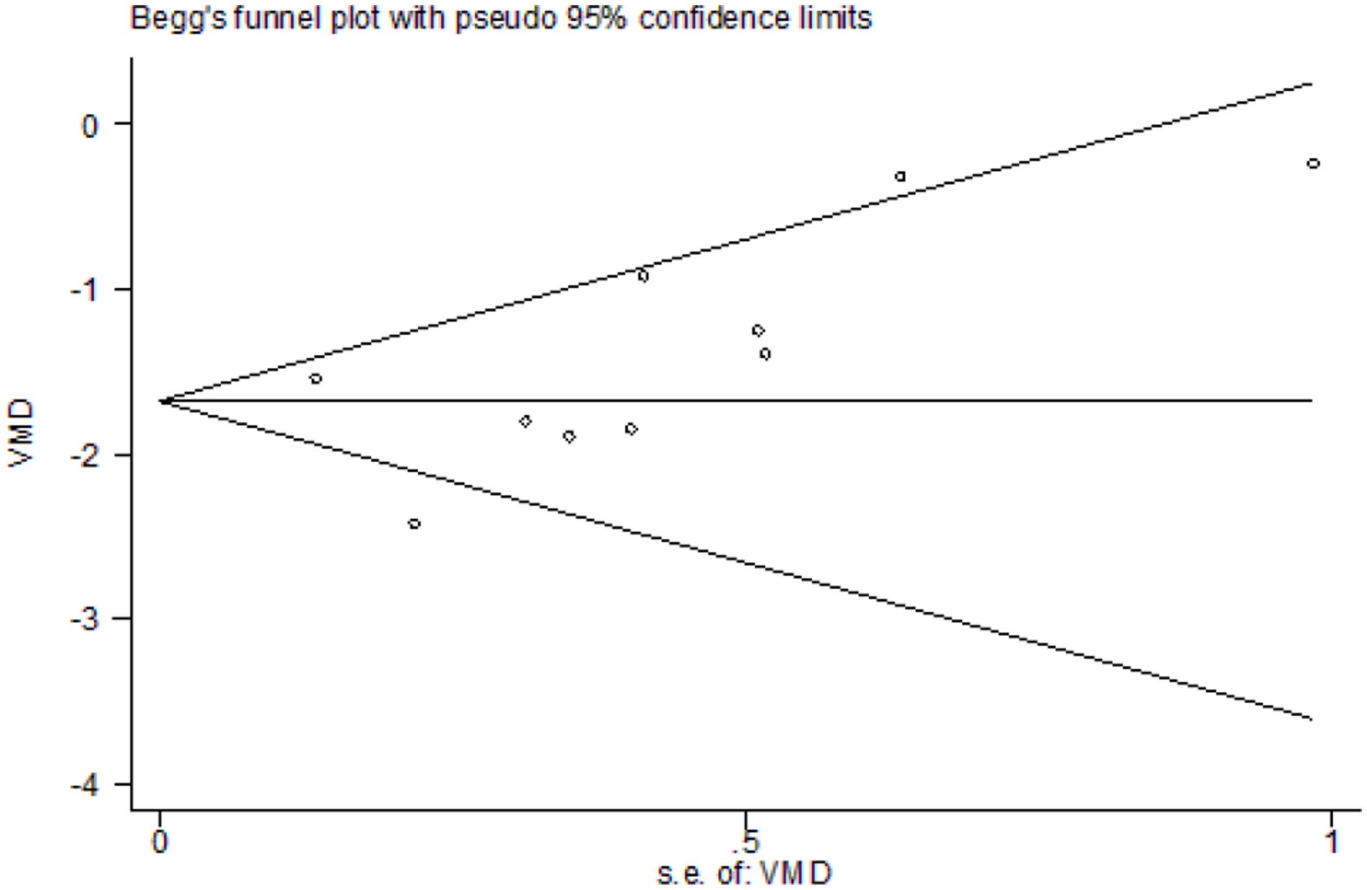
Begg’s funnel plot of publication bias.

**Figure 16:**
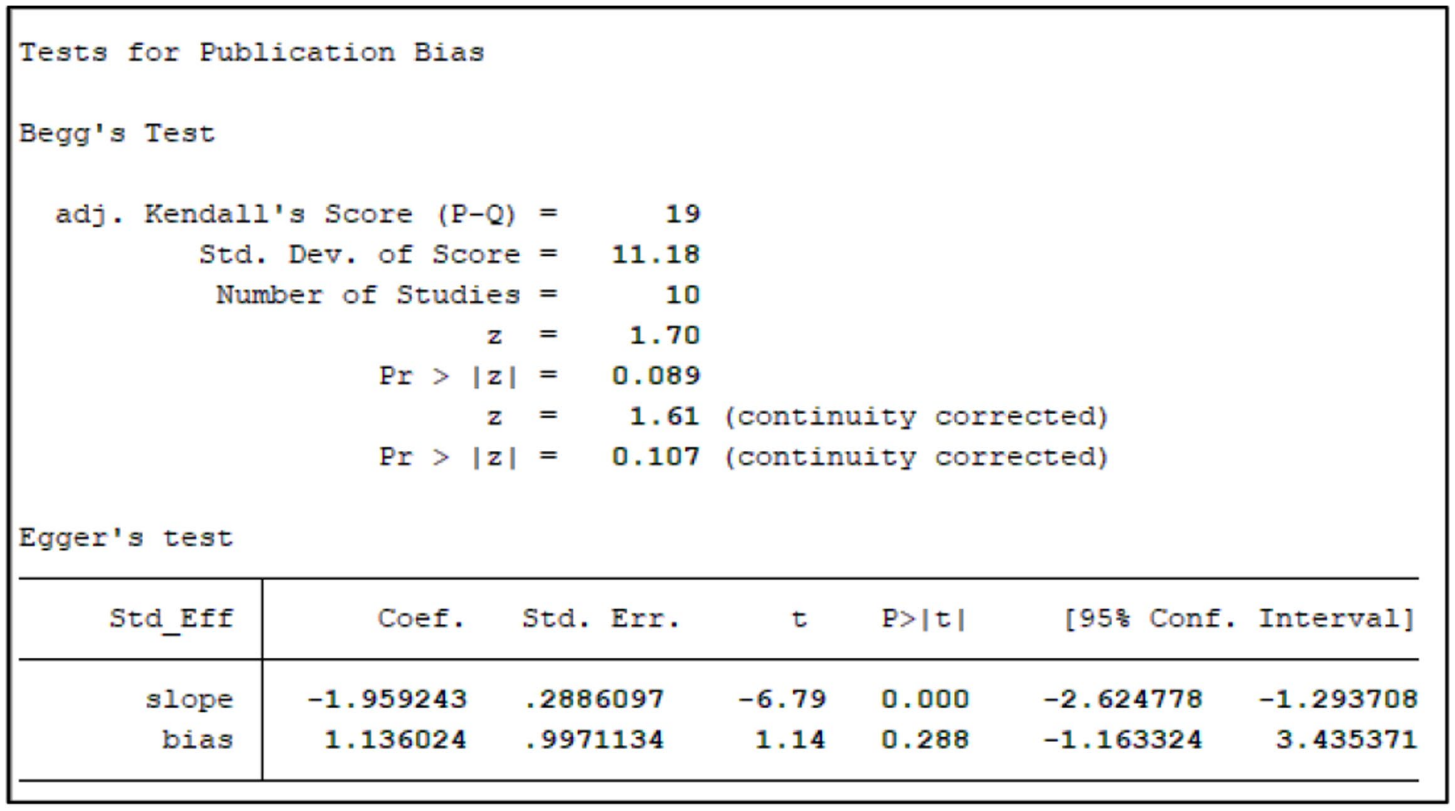
Tests for publication bias.

We assessed publication bias for the primary outcome (effective rate) using Begg’s funnel plot and Egger’s linear regression test (Figure 15). Visually, the funnel plot appeared roughly symmetrical. Statistical tests further confirmed the absence of significant publication bias (Begg’s test, *p* = 0.272; Egger’s test, *p* = 0.053; Figure 16). This suggests that the findings of this study are unlikely to be substantially influenced by publication bias.

### 3.11 GRADE assessment

Table 2 presents a summary of the evidence quality assessment for outcome measures evaluating the effects of BLT. Based on the GRADE framework, the certainty of evidence for outcomes in this review varied from moderate to very low. Critical outcomes including effective rate and PHN incidence were assessed as moderate, limited mainly by serious risk of bias. Evidence supporting pain reduction (VAS) was rated low due to significant heterogeneity and imprecision. Safety data (adverse reactions) and the mechanistic biomarker TH17/Treg were graded as very low, primarily constrained by imprecision, suspected publication bias, and indirectness for the surrogate outcome.

**Table 2:** GRADE quality of evidence assessment of outcome indicators for the included studies.

Outcome/no. of studies	Design	Quality assessment					No. of patients	Relative (95% CI) absolute	Quality	Importance
		Risk of bias	Inconsistency	Indirectness	Imprecision	Publication bias				
Effective Rate (*n* = 13)	Randomized trials	Serious ^a^	Not serious	Not serious	Not serious	Not detected	1,020	OR = 4.51 [2.89, 7.04]	Moderate	Critical
VAS (*n* = 10)	Randomized trials	Serious ^a^	Serious ^b^	Not serious	Serious ^d^	Not detected	682	MD = −1.57 [−1.93, −1.21]	Low	Critical
TVC (*n* = 6)	Randomized trials	Serious ^a^	Serious ^b^	Not serious	Serious ^d^	Suspected ^e^	440	MD = −1.04 [−1.65, −0.43]	Low	Important
TVF(*n* = 8)	Randomized trials	Serious ^a^	Not serious	Not serious	Not serious	Suspected ^e^	607	MD = −1.58 [−1.79, −1.38]	Moderate	Important
TVD (*n* = 4)	Randomized trials	Serious ^a^	Serious ^b^	Not serious	Serious ^d^	Suspected ^e^	276	MD = −2.53 [−3.82, −1.23]	Low	Important
Incidence of PHN (*n* = 10)	Randomized trials	Serious ^a^	Not serious	Not serious	Not serious	Not detected	808	OR = 0.23 [0.15, 0.35]	Moderate	Critical
Adverse reactions (*n* = 7)	Randomized trials	Serious ^a^	Not serious	Not serious	Serious ^d^	Suspected ^e^	514	OR = 0.66 [0.31, 1.38]	Very Low	Important
TH17/Treg (*n* = 3)	Randomized trials	Serious ^a^	Not serious	Serious ^c^	Serious ^d^	Suspected ^e^	242	MD = −0.04 [−0.08, 0.00]	Very Low	Exploratory

GRADE: Grading of Recommendations Assessment, Development, and Evaluation; VAS: Visual Analogue Scale; TVC: Time to Vesicle Cessation; TVF: Time to Crust Formation; TVD: Time to Scab Detachment; CI: Confidence Interval; MD: Mean Differences.

a *Most RCTs at high risk of bias due to methodological limitations*.

b Evidence of statistically significant interstudy heterogeneity.

c Surrogate outcome.

d Wide confidence intervals.

e Insufficient number of included studies and the funnel plot is slightly asymmetrical.

## 4 Discussion

### 4.1 Summary of results

This systematic review evaluated BLT for HZ symptom management. Analyses demonstrated bloodletting’s superiority over drugs in overall effectiveness, pain relief (VAS reduction), accelerated lesion healing, PHN prevention. Safety profiles were comparable, though TH17/Treg modulation did not differ significantly. However, methodological limitations (e.g., high heterogeneity, suboptimal blinding) preclude definitive conclusions.

### 4.2 Limitations

This systematic review has several noteworthy limitations that warrant careful consideration.

First, all studies originated from China restricts the generalizability of our findings, as regional variations in clinical practice and publication bias may influence outcomes. Notably, acupuncture-related RCTs from China demonstrate a significantly higher proportion of positive results compared to Western counterparts, suggesting potential publication bias favoring favorable outcomes.

Second, the overall methodological quality of the included trials was suboptimal. Notably, none of the studies implemented double-blinding, although three employed assessor blinding. Furthermore, most trials inadequately described allocation concealment, and four studies exhibited attrition bias due to incomplete intention-to-treat analysis of dropouts. The possibility of selective reporting bias could not be ruled out in most studies, as preregistered protocols were unavailable, suggesting that some negative outcomes might have gone unreported. These limitations may collectively introduce systematic bias, thereby undermining the generalizability and practical applicability of the findings. The potential impact of attrition on the pooled effect estimates warrants further investigation. In open-label studies, the observed therapeutic effects, particularly for subjective outcomes such as pain relief, are likely to include a considerable level of placebo and expectation effects. As a result, the aggregated results may overestimate the actual benefits of BLT, since the available evidence might be disproportionately skewed toward positive results.

Third, the robustness of our meta-analysis is constrained by significant heterogeneity in study design. Specifically, bloodletting protocols varied substantially in technique, volume, and treatment duration, while control groups received diverse drug regimens with inconsistent treatment courses. Notably, 14 of the 16 included studies failed to incorporate guideline-recommended neuropathic pain medications, and one even omitted antiviral therapy. In subsequent research, more attention should be paid to formulating medication plans in a more standardized manner in accordance with the treatment guidelines. Furthermore, the robustness of our conclusions is constrained by several methodological limitations. The evidence base lacks long-term follow-up data (>6 months), which is critical for definitively assessing the preventive effect of BLT on PHN, as this condition is defined by pain persisting beyond 90 days. Additionally, key mechanistic biomarkers such as the TH17/Treg ratio were only reported in three studies, resulting in insufficient statistical power to draw meaningful conclusions from these outcomes. Although some studies reported improvements in quality-of-life metrics, the data remained too sparse for quantitative synthesis, thereby limiting our ability to assess these patient-centered outcomes systematically.

Finally, the selection of clinical effective rate as the primary outcome in this review reflects its predominant reporting frequency in Chinese RCTs. Nevertheless, this metric lacks universal recognition due to inconsistent definitions of “clinical effectiveness” across studies (54). Consequently, meta-analytic findings derived from effective rates warrant cautious interpretation. Furthermore, in interpreting the pain reduction (MD: −1.57 on the VAS), it should be noted that while our pooled estimate exceeds the commonly cited MCID threshold for acute pain, the MCID itself can vary across populations and contexts. Moreover, as a meta-analysis, we could only synthesize mean differences rather than the proportion of individual patients achieving a clinically important improvement. Future primary studies should incorporate patient-centered anchors to directly assess this proportion, which would provide more robust evidence regarding the clinical meaningfulness of BLT.

### 4.3 Implications for practice and research

To strengthen the evidence, high-quality RCTs are essential before definitive conclusions can be drawn. Researchers designing prospective RCTs on BLT for herpes zoster should adhere to the CONSORT 2010 guidelines (55, 56) and preregister all trials per ICMJE recommendations (57) prior to participant enrollment.

Adequately powered studies with sufficiently large sample sizes are critical to ensure statistical robustness. Methodological rigor must include strict blinding procedures, allocation concealment with detailed documentation of randomization processes, and extended follow-up periods to enhance generalizability. Although complete blinding is challenging with this intervention, protocols should aim to blind both patients and assessors whenever feasible.

Standardized protocols should be established for both intervention and control groups, particularly defining optimal bloodletting techniques, treatment frequency, session-specific blood volume, and duration, with explicit investigation into dose–response relationships between blood volume and pain relief. Outcome measures ought to incorporate objective biomarkers alongside validated patient-reported outcomes such as the Pittsburgh Sleep Quality Index (PSQI) and quality-of-life (QoL) metrics, thereby enabling comprehensive clinical evaluation.

Although BLT shows potential benefits, current evidence remains insufficient to recommend routine clinical application given methodological flaws and possible placebo effects in open-label trials. A more balanced viewpoint should acknowledge the potential influence of expectation and contextual effects. Comparative effectiveness research against established treatments (e.g., pharmacotherapy, acupuncture, cupping) is also needed to position BLT within integrated therapeutic strategies for HZ.

## 5 Conclusion

Based on currently available low-quality evidence, bloodletting therapy (BLT) appears to be associated with certain short-term benefits in acute herpes zoster, including symptomatic relief, accelerated lesion healing, and potential prevention of postherpetic neuralgia, alongside an acceptable safety profile. However, these results must be interpreted with caution due to considerable methodological heterogeneity and a geographically limited evidence base consisting exclusively of studies from China. Consequently, the generalizability of findings to non-Chinese populations remains uncertain, and BLT cannot currently be recommended for routine use in neurological practice. These limitations underscore the necessity for rigorously designed, internationally validated trials with standardized treatment protocols to objectively assess efficacy and to establish its potential role in clinical settings.

## Data Availability

The original contributions presented in the study are included in the article/Supplementary material, further inquiries can be directed to the corresponding authors.
