# Supplementary material for: Efficacy and safety of bloodletting therapy for acute herpes zoster: a systematic review and meta-analysis
Source: Front Neurol. 2025 Oct 13;16:1674245. doi: 10.3389/fneur.2025.1674245 (PMC12554594; doi:10.3389/fneur.2025.1674245)
Supplement: Supplementary file 1 [file Data_Sheet_1.PDF]

search strategy

Pubmed:

#1 Acupuncture Therapy [MeSH Terms]

#2 Acupuncture [Title/Abstract]

#3 Cupping [Title/Abstract]

#4 Bloodletting [Title/Abstract]

#5 Three-edged needle [Title/Abstract]

#6 triangular needle [Title/Abstract]

#7 Blood-letting Therapy [Title/Abstract]

#8 pricking [Title/Abstract]

#9 phlebotomy therapy [Title/Abstract]

#10 #1 OR #2 OR #3 OR #4 OR #5 OR #6 OR #7 OR #8 OR #9

#11 Herpes Zoster [MeSH Terms]

#12 Zona [Title/Abstract]

#13 Zoster [Title/Abstract]

#14 Shingles [Title/Abstract]

#15 #11 OR #12 OR #13 OR #14

#16 Randomized Controlled Trial [Publication Type]

#17 Controlled Clinical Trial [Publication Type]

#18 Randomized [Title/Abstract]

#19 Randomly [Title/Abstract]

#20 Trial [Title/Abstract]

#21 #16 OR #17 OR #18 OR #19 OR #20

#22 #10 AND #15 AND #21

Embase

#1 acupuncture:ti,ab,kw OR 'acupuncture therapy':ti,ab,kw OR cupping:ti,ab,kw OR

bloodletting:ti,ab,kw OR 'three-edged needle':ti,ab,kw OR 'triangular needle':ti,ab,kw OR 'blood-letting therapy':ti,ab,kw OR pricking:ti,ab,kw OR 'phlebotomy therapy':ti,ab,kw

#2 'herpes zoster':ti,ab,kw OR zona:ti,ab,kw OR zoster:ti,ab,kw OR shingles:ti,ab,kw OR 'postherpetic neuralgia':ti,ab,kw

#3 'randomized controlled trial':ti,ab,kw OR 'controlled clinical trial':ti,ab,kw OR randomized:ti,ab,kw OR trial:ti,ab,kw OR randomly:ti,ab,kw

#4 #1 AND #2AND #3

中国知网（CNKI）:

SU= 带状疱疹 + 带状疱疹急性期 + 带状疱疹后遗神经痛 + 带状疱疹神经痛 + 带状疱疹后遗症 + 蛇串疮 + 缠腰火丹 AND SU=刺络拔罐 +梅花针 + 放血 + 刺络 + 刺血 + 三棱针 + 拔罐 +火罐 AND SU=随机对照 + 随机对照试验 + 随机 +对照 +对比 +临床研究 + 临床试验 +临床观察

万方（Wan Fang database）:

检索表达式: 主题:(带状疱疹 or 带状疱疹急性期 or 带状疱疹后遗神经痛 or 带状疱疹后遗症 or 蛇串疮 or 缠腰火丹) and 主题:(刺络拔罐 or 梅花针 or 放血 or 刺络 or 刺血 or 三棱针 or 拔罐 or 火罐) and 主题:(随机对照 or 随机对照试验 or 随机对照研究 or 随机 or 对照 or 对比 or 临床研究 or 临床观察 or 临床试验)

维普(VIP Chinese Science):

M=（带状疱疹 OR 带状疱疹急性期 OR 带状疱疹后遗神经痛 OR 带状疱疹神经痛 OR 带状疱疹后遗症 OR 蛇串疮 OR 缠腰火丹） AND M=（刺络拔罐 OR 梅花针 OR 放血 OR 刺络 OR 刺血 OR 三棱针 OR 拔罐 OR 火罐） AND M= （随机对照 OR 随机对照试验 OR 随机 OR 对照 OR 对比 OR 临床研究 OR 临床试验 OR 临床观察）
